# Entangled brains and the experience of pains

**DOI:** 10.3389/fpsyg.2024.1359687

**Published:** 2024-03-15

**Authors:** Valerie Gray Hardcastle

**Affiliations:** Institute of Health Innovation, Northern Kentucky University, Highland Heights, KY, United States

**Keywords:** pain, brain, neural correlate, reduction, navigation, adaptive, multiplex

## Abstract

The International Association for the Study of Pain (IASP) revised its definition of pain to “an unpleasant sensory and emotional experience.” Three recent recommendations for understanding pain if there are no clear brain correlates include eliminativism, multiple realizability, and affordance-based approaches. I adumbrate a different path forward. Underlying each of the proposed approaches and the new IASP definition is the suspicion that there are no specific correlates for pain. I suggest that this basic assumption is misguided. As we learn more about brain function, it is becoming clear that many areas process many different types of information at the same time. In this study, I analogize how animal brains navigate in three-dimensional space with how the brain creates pain. Underlying both cases is a large-scale combinatorial system that feeds back on itself through a diversity of convergent and divergent bi-directional connections. Brains are not like combustion engines, with energy driving outputs via the structure of the machine, but are instead more like whirlpools, which are essentially dynamic patterns in some substrates. We should understand pain experiences as context-dependent, spatiotemporal trajectories that reflect heterogeneous, multiplex, and dynamically adaptive brain cells.

## 1 Introduction: defining pain

“All we get are a few specks of time where any of this actually makes any sense.”Joy Wang

*Everything Everywhere All at Once*

Intuitively, we think of pains as our bodies’ response to some sort of damage. But in 2020, the International Association for the Study of Pain (IASP) revised its definition of pain such that pain is (only) “*an unpleasant sensory and emotional experience* associated with, or resembling that associated with, actual or potential tissue damage” (Raja et al., 2020, p. 1977, italics mine).^1^ “Pain” now is just the conscious sensation of pain. Our best scientific account of pain has been divorced from what we think of as its typical cause.

This perspective makes the scientific study of pain challenging, to say the least. Three recent recommendations for understanding pain if there are no clear brain correlates include (1) promoting some version of eliminativism (Corns, 2020; Liu, 2023), (2) reviving multiple realizability and family resemblance models (Borg et al., 2021; Coninx, 2023; Coninx et al., 2023b; Serrahima and Martínez, 2023), and (3) suggesting an intersubjective or affordance-based approach (Oliver, 2022; Coninx et al., 2023a; Fulkerson, 2023).

Here, I adumbrate a different path forward. Underlying each of the above-proposed approaches is the suspicion that there are no specific neural correlates for pain. We do not have an area in the brain devoted to pain processing in the same way that we have a visual pathway, for example. In this article, I will suggest that this basic assumption is incorrect, or, better, misguided. Indeed, as we learn more about brain function, it is becoming clearer that many areas process many different types of information at the same time. The fact that there might be no specific correlates for pain that do not overlap with other sensations (*cf.*, Coninx, 2023) is not indicative of anything unusual about how brains function.

Historically, we have taken a reductive approach to understanding the brain. Consider Nagel’s description of how we should explain headaches: once “the detailed physical, chemical, and physiological conditions for the occurrence of headaches are ascertained … [then] an explanation will have been found for the occurrence of headaches” (Nagel, 1961, p. 366). Taking this sort of reductive explanatory approach means that we learn about brain function through decomposing brain areas into sets of individual cells and then to their individual reactions. We slice the brain into smaller and smaller pieces and then articulate how all these pieces connect to one another and interact as a larger whole. Then, voila! We have explained a brain phenomenon.

But recent work suggests that brain structures, both big and small, are fundamentally interwoven. I shall describe this alternative conceptualization of brain organization and function using a brief history of understanding how brains navigate in space as an example (Mallory et al., 2021; Maisson et al., 2022). My primary point will be that a variety of brain areas support multiple adaptive behaviors and internal representational schemes. In other words, many brain areas that were once thought to do just one thing turn out to support a wide range of functions, and they do so simultaneously.

I shall use this approach to articulate an alternative way of understanding pain. My wider conclusion, however, will be that philosophical intuitions regarding conscious pains and pain processing (or any sort of brain-related functions) are probably best to be avoided. Instead, how our brains do what they do is rooted deep in our evolutionary history, and their functions do not reflect our linguistic divisions or human conventions. Carving nature at its actual joints will require letting go of many contemporary philosophical categories (*cf.*, Westlin et al., 2023). None of the three putative ways to understand pain are likely to be correct. We do have specific neural correlates for pain and pain experiences, but they are not what one might intuitively imagine them to be.

## 2 Philosophical agreement with the IASP

The three approaches mentioned above—eliminativism, multiple realizability, and affordances—all essentially accept the IASP’s perspective without question. They all agree that pain as a rich and complex experience is not reducible (or fully reducible) to underlying brain activity. For example, in her new book promoting eliminativism, Corns (2020) argues that pain is not a natural kind because it cannot be scientifically “projected”. The cellular interactions that determine instances of pain differ among individuals; therefore, they “undermine … explanations of pain types or pain as such” (p. 141). Pain cannot be a scientific object of study because its instantiation in brains is not constant across individuals or within individuals over time.

Similarly, Coninx agrees that pain experiences across individuals or even within an individual across time are disunified. Nevertheless, as a proponent of a family resemblance approach, she suggests that “[glossing] over differences between pain cases can prove useful under certain conditions for certain scientific purposes” (Coninx, 2023, p. 186). Even though pain may not be a natural kind, we could use the “resemblance relations” among the neural patterns for pain to create broad but serviceable generalizations that could be used in science or medicine to achieve particular ends, like developing effective treatments. She suggests that in this way, pains could loosely form a sort of “phenomenal kind” (p. 180).

Finally, affordance-based approaches to pain also agree that pain is not (just) a type of brain activity that refers to a perceived bodily condition. For example, Oliver (2022) explains that pain states are experienced from a first-person point of view that is embedded in a rich sociocultural environment and that we ascribe meaning to pain experiences in virtue of our respective communities. Pain is “about the interdependent way multidimensional biopsychosocial factors are of concern to a subject” (p. 18). That is, “pain” refers to these specific integrated experiences of sensation, emotion, and interpretation/evaluation in a particular body as it exists in a specific environment through which the person perceives that they can do/see/experience/think certain things (see also Coninx et al., 2023a). Pain is much more than mere brain activity; we would need to appeal to the relevant aspects of the body and environment to give a proper explanation of a pain experience. Neural correlates alone could never underpin a complete theory (see also Hutto and Myin, 2013).

However, while these affordance-based approaches agree with the other two that pain is complex, they disagree that “pain” refers to dissociable cognitive, affective, and physical aspects (this dissociation then either prevents scientific reduction, as Corns claims, or supports loose generalizations, as Coninx claims.) Instead, the multidimensional biopsychosocial factors exist as a complex whole in an embodied mind. A proper nonreductive science of embodied pain might, thus, be possible (see also Coleman, 2020; Cormack et al., 2022).

Even though the three philosophical approaches all differ on what pain being irreducible to brains implies, they, along with the new IASP definition, all agree that there is no easy one-to-one correspondence between any set of pains and identifiable and consistent brain activity. Many recent neuroscientific investigations into pain also support this perspective: there appears to be a range of different neural structures in different locations across the brain that are involved in pain processing (Apkarian et al., 2005; see also Kucyi and Davis, 2014; Bastuji et al., 2016), and yet none of them seem either necessary or sufficient for the experience of pain (Apkarian, 2017). Additionally, none of these areas are identified with pain exclusively; they are also associated with itch, touch, heat, and difficulty breathing (Evans et al., 2002; Iannetti and Mouraux, 2010; Legrain et al., 2011; Liberati et al., 2016; Dong and Dong, 2018). In the scientific literature, there has been at least the suggestion that there are no underlying mechanisms specific to the experience of pain, nor any clear pattern of activity for it across the brain. Even from science’s point of view, it appears more likely than not that pains are not a natural kind (although Bateu, 2020 and Djordjevic, 2023 suggest a different perspective).

It is easy to see how the IASP reached its revised definition and how many philosophers are coalescing around the idea that pain experience is not a proper object of brain study. Nevertheless, I believe a different (and better) approach is possible. This approach starts by embracing the complexity of pain and the brain in all its glory.

## 3 A different approach

Perhaps more important than the challenge of the apparent irreducibility of pain is that Corns’s, Coninx’s, and Oliver’s approaches to understanding pain ignore or overlook the question of why the quality of pain is the explanatory target in the first place. Regardless of approach, there is agreement that being in pain is a complex state, one that involves a variety of qualia – negative affect, motivational states, sensation, judgments – along with a variety of psychological processes: memory, attention, mood, alertness (see also Borg et al., 2021; Liu, 2023). Why set all this aside as irrelevant and focus on what appears to be only one aspect of pain?^2^ The IASP’s definitional revision to remove pain from its physical substrate means that their new perspective on “pain” misses much of what pains actually are. We must recognize and address the full complexity of pain, including the experience of pain, if we are going to advance the science of pain.

If we take seriously the idea that we need to include all the facets of pain in any scientific theory of pain, the first thing to note is that bodily injury drops out as fundamental to pain. Even though acute injury-based pain is the model for most animal-based pain research and pain theorizing, there are simply too many types of non-injury-related pains to have acute injury be the paradigmatic cause of a pain. Indeed, there is a range of well-defined pain-related disorders. Aside from the challenge of chronic pain, there are also allodynia, arthritis, complex regional pain syndrome type 1, causalgia, chronic fatigue syndrome, fibromyalgia, headaches, irritable bowel syndrome, neuropathic pain, orchialgia, phantom limb pain, radicular pain, temporomandibular disorder, and trigeminal neuralgia, among others. There is also a range of headaches, referred pains, neuromas, and cancer pains, as well as things like menstrual pain (*cf.*, Serrahima and Martínez, 2023), that have no obvious “injury” cause and often no obvious cause at all.

IASP has recognized this issue and has divided pain into three broad categories: nociceptive, neuropathic, and nociplastic. Nociceptive pain refers to what we normally think of as injury-based pain; it includes all pains arising from tissue damage. Neuropathic pain arises from damage to the nerves themselves – things like sciatica. Nociplastic pain means something like “altered nociception;” a pain for which there is no (obvious) disease, lesion, or tissue damage (IASP, n.d.; see also Buldys et al., 2023). Identified only in 2016, we currently have no clear idea what nociplastic pain is, other than a painful condition that has no identifiable cause.

I am mentioning the wide range of pains to underscore that pain is indeed complex and multifarious and may be only roughly unified in terms of its sensation. Explanations of pain could very well be complex, multifarious, and only weakly unified as well. Recent event-related potential (ERP) research provides a nifty example of how one might (start to) build a theory of such complex phenomena.

In ERP studies, multiple very sensitive electrodes that can measure the electrical impulses that are primarily driven by neural interactions (the EEG waves) are placed on the scalp. If research subjects experience painful stimuli, such as thermal heat on their skin, their brains notice, interpret, and respond to the stimuli. Averaging time-locked brain signals across the skull over multiple similar stimuli produces signature activity patterns, which reflect the brain’s response to that sort of stimulus. Sophisticated analytical techniques, combined with known brain structures, allow for some internal localization of the origin of the brain responses. In comparison to fRMI scans, ERP studies provide for better temporal resolution but poorer spatial resolution of stimulus-evoked brain responses. We now know that brains can respond across a variety of frequencies to external stimuli, even when responding to the same stimulus over time. Combining stimuli duration and intensity with brain response duration and frequency as well as location estimates can paint a compelling picture of what the brain is doing with information it is receiving from the external world.

A group of scientists working together across several laboratories recently reported that they have identified brain responses that appear keyed to the transition of a painful stimulus to a pain percept. By varying the intensity and the duration of the painful thermal stimulus and then comparing the various localized neural responses as recorded across the scalp with each other and to subjective pain reports, the scientists could demonstrate that both responses reflected subjective pain ratings for duration and intensity. In particular, the sensation of incidental but extended thermal pain (or rather, the report of such a sensation) co-occurred with a low-frequency waveform (< 1 Hz) originating in the insula and the anterior singular cortex (the medial pain system) and an alpha-band (8–13 Hz) desynchronization in the sensorimotor cortex (the lateral pain system).^3^ The two waveforms were coupled with each other, with the alpha oscillations fluctuating with the low-frequency waveform (Wang H. et al., 2023). This sort of coupling suggests that the underlying brain structures are responding simultaneously to the same inputs and that whatever is going on inside the brain is distributed and complicated. Further, because the duration of the coupling was correlated with the duration of the pain perception, the waveforms also index the experience of pain. All these data suggest that multiple brain regions are involved in converting stimuli to perceptual awareness of the stimuli.

Additionally, the size of the recorded waves over the insula and the anterior singular cortex varied by reported stimulus intensity. This result aligns with previous EEG and fMRI studies (e.g., Atlas et al., 2014; Woo et al., 2015; Tiemann et al., 2018), which supports the idea that these brain waves are correlated with the brain translating stimulus intensity into concomitant sensations of pain intensity. These responses were in the areas that process the salience of stimuli, especially where pain is concerned (*cf.*, Guo et al., 2020). These data also dovetail with data from implanted EEG electrodes in patients being monitored for epileptic foci who experienced a range of durations and intensities of thermal pain under controlled conditions (Caston et al., 2023). We would expect a tight correspondence between pain intensity and pain salience. Thus, the waves in the insula and the anterior singular cortex could reflect the impact the brain’s estimation of salience has on the perceived intensity of a stimulus (see also Liberati et al., 2018).

I do not intend to lean too heavily on these studies to support any particular conclusion about the substrates of pain experiences, no matter how elegant, but I do intend to use them to suggest a different approach to understanding the brain bases of pain, one that embraces the complexity of brain responses as well as the complexity of pain – and one that looks at more than brain regions and their gross responses to stimuli. I suggest that what makes up a sensation is much more complicated and subtle than philosophers have previously assumed. As explained below, brains are not like combustion engines, with energy driving outputs via the structure of the machine, but are instead more like whirlpools, which are essentially dynamic patterns in some substrates. To reach this conclusion, I shall analogize studies of how the brain navigates in three-dimensional space with how the brain creates pain.

## 4 Animal navigation

Just like pain processing, navigation in a three-dimensional environment is a complex process. To be useful for the organism, it must combine internal goals and desires with external data and motor planning in real time. What the brain’s navigational codes are and how they are implemented have been continuously investigated by neuroscientists for over 50 years, going back to when O’Keefe and Dostrovsky (1971) first identified spatially tuned cells, dubbed “place cells,” in the hippocampus. These cells increased their average firing rates as the animal approached the places to which they were “tuned,” thereby creating a “grid map” that represented the navigable environment around an animal. Over the next half a century, additional spatial cells that were tuned to other animal-environment relationships were also discovered, e.g., allocentric head direction cells (Taube et al., 1990), allocentric border cells (Savelli et al., 2008; Solstad et al., 2008), egocentric boundary cells (Wang et al., 2018; Hinman et al., 2019), etc. At first, these types of cells were only found in and around the hippocampus, which led some to conclude that the hippocampus contained each animal’s cognitive map of its world, which in turn supported the animal’s movement in its environment. Perhaps, the hippocampal formation could be the navigation center of the brain (see, e.g., O’Keefe and Nade, 1978).

However, not surprisingly, that supposition was too facile, and, over time, scientists have identified many navigational tuning cells throughout the brain, including in the brainstem, cerebellum, and cortex. Indeed, navigational processing seems to be widely distributed throughout the brain. Of course, this makes sense, given that animals must tap their sensory systems, their memory systems, and their motor system to be able to move freely and successfully in complex three-dimensional spaces. At the same time, researchers also learned that the codes that brains used to navigate with were extremely dynamic; they did much more than just passively encode 3-D spatial relationships (see Maisson et al., 2022 for a review). Instead, navigational processes seem to be fundamentally integrated into all the other decision-making that animals must undertake to move about in the world in real time. For instance, the medial temporal cortex in mice integrates sensory inputs, the movements of their eyes and head, and a myriad of other cues to generate a map of landmarks in space (Mallory et al., 2021). Hardcastle et al. (2017) determined that these sorts of neural codes are “*highly multiplexed*,” “*heterogeneous*,” “and “*dynamically adaptive*” (italics mine). Importantly, this complex structure can support a degree of computational flexibility that allows animals to respond to their ever-changing bodily needs in real time as they navigate across complex landscapes (see also Pessoa et al., 2021).

This sort of theoretical advance reinforces the idea that our brains do not comprise a cortex, doing one set of tasks, riding on top of more primitive subcortical regions, doing a different set of tasks. Instead, the brain consists of widely “distributed and entangled” networks (see Pessoa, 2022; Westlin et al., 2023). That is, the brain is not an assemblage of neural circuits but a large-scale combinatorial system that feeds back on itself through a diversity of convergent and divergent bi-directional connections. The moral of this story is that we should understand what brains are in the same way that meteorologists understand whirlpools (or hurricanes) – as dynamic, context-dependent, spatiotemporal trajectories (*ibid.*, pp. 227–228).

Fortunately, as scientists were beginning to realize that animal navigation was even more complex and distributed than originally envisioned, they were also devising new and better ways to analyze brain activity. They moved from the tuning curves of yore, which were simple peristimulus time histograms, to representational similarity analyses, or “RSA.” In brief, RSA makes pairwise comparisons between conditions of an experimental intervention, using distance matrices to capture the similarity of a given measure for neural activity, behavior, or model output. One can then use these sets of comparisons to analyze whether and how the so-called representational distance matrices, or “RDMs,” vary across contexts, species, regions, models, and so on (*cf.*, Nili et al., 2014). Additionally, new holographic optogenetic techniques, operating on a millisecond timescale (Adesnik and Abdeladim, 2021), permit more realistic representations of individual neuronal interactions. With this technique, researchers can also analyze more than the outputs of a small set of single cells in a brain area. For just one example, Allen et al. (2019) recorded neuronal activity from approximately 24,000 cells simultaneously across 34 cortical and subcortical regions. These recordings demonstrated that it takes only approximately 300 milliseconds for salient sensory stimuli to propagate across the entirety of a rat’s brain.

In neuroscience’s early days, scientists believed that individual neurons had just one primary task, which determined their coding properties. And these properties changed little over time. For example, an individual head direction cell would encode the direction of the head when it was pointing this way but no other (*cf.*, Taube et al., 1990). Neuroscience’s job was to functionally identify all the different types of neurons involved in each deconstructed brain process. We can see this approach in our early understanding of vision: simple cells fed into complex cells, which then built up into more hierarchies and more complex hierarchies (*cf.*, Hubel and Wiesel, 1962).

But today, we have more complex statistical tools that we can use to analyze what cells are responding to, which gives us a different perspective on how brains do their work. Instead of encoding just a single property, we now know that most “navigation” cells simultaneously encode head direction, motion, and location (Sargolini et al., 2006); that is, they are *multiplex*. But even though each of these cells is sensitive to virtually all the features important to the animal moving across its environment, what they are sensitive to differs across cells. It would not be surprising if each cell were to have its own unique combination of informational sensitivities. In other words, neuronal responses are also *heterogeneous* (Hardcastle et al., 2017).

Additionally, we have learned that what cells respond to is not the same across time or conditions. The old view was that if a navigational cell encoded position in one way, it will always encode for position and for position in exactly that way. Researchers have described how animal brains navigate in terms of an internal two-dimensional latitude-longitude coordinate map coupled with an internal compass (*cf.*, Moser and Moser, 2016). However, we now know that if an animal is actively navigating, cell responses become increasingly precise. If it is navigating toward a reward, the neurons record where the reward is more accurately. On the other hand, if an animal is moving slowly and randomly, it responds to location and space less precisely (Hardcastle et al., 2017). In other words, brain cells are adaptive: they become more specific when responding to the more important parts of their environment. The resolution of neurons can change depending on how precise the animal needs it to be in that moment. That is, neural responses are *dynamically adaptive*. And furthermore, multiplex, heterogeneous, and dynamically adaptive brain cells open up new ways of envisioning brain function, as random combinations of variables create a broader space in which brains can learn.

In addition to highlighting the complexity of brain response supporting animal navigation, it should also be indicated that a multiplex, heterogeneous, and dynamically adaptive way of understanding brain function and organization does not belie reduction. No one is claiming that because neurons respond dynamically in a complex manner depending upon animal needs, or because no single area or type of neuron appears to respond to only navigational tasks and nothing else, we cannot reduce animal navigation to brain activity. Instead, researchers are spending their careers trying to map out exactly how brains respond to complex navigational challenges in real time, how neurons work together across regions to move animals to food and shelter, away from foes, and toward mates, depending on their specific hierarchy of needs at that moment.

What if pain is expressed in the same way in brains? What if pain is a highly multiplexed, heterogeneous, and dynamically adaptive process of response to adverse stimuli? Just as we do not understand whirlpools by virtue of the location and directional movement of individual droplets of water, perhaps we should not understand pain in terms of brain areas and static neuronal responses. Instead, we want to know how different brain structures and responses “unfold temporally” to support pain experiences and pain behaviors (Pessoa, 2022, p. 227; see also Westlin et al., 2023). In this case, neuroscientists would strive to understand pain by describing “the *joint state* of brain regions and how it changes;” that is, by describing the brain’s “spatiotemporal trajectories” associated with pain processes and responses (*ibid.*, p. 228, italics in the original). This approach could keep all the fantastic individuality and complexity of pain but also allow for its reduction to the brain.

## 5 Pain as heterogeneous, dynamically adaptive, and highly multiplexed neural responses

The idea of neural correlates of pain being at least heterogeneous is not new. Melzack (1999) conceived a “pain neuromatrix” over two decades ago. This matrix references an interconnected network of neural areas that support pain processing, as opposed to a single pain region or pathway in the brain. This network generates distinctive patterns of activation that correspond to different pain experiences (Melzack, 2001). It is divided anatomically and functionally into medial and lateral ascending pathways. The medial pathway processes the affective dimensions of pain via a circuit traveling from the parabrachial nucleus to the amygdala and then to the prefrontal cortex and anterior cingulate cortex. The lateral ascending pathway supports the sensory/discriminative dimensions of pain and is composed of the thalamus, somatosensory cortex, and insular cortex. It is worth noting that this division is here for ease of discussion. It is quite clear that the divisions between affective and sensory information are rather artificial and that there is quite a bit of crosstalk between the two ascending pathways (*cf.*, Giesler et al., 1981; Apkarian, 2012.) There is also a descending pathway that starts in the prefrontal cortex and travels back through the anterior cingulate cortex, amygdala, hypothalamus, and periaqueductal gray, which modulates pain signals in brainstem nuclei that project to the spinal cord (Yao et al., 2023).

Importantly, this network can be influenced by attention and stress, among other states (Tracey et al., 2002; Tracey and Mantyh, 2007; Ploner et al., 2011). How they influence pain perceptions varies across individuals and within the same individual over time. Differential reactions to the same painful stimulus appear to be keyed to differential activation in the dorsolateral prefrontal cortex (Crawford et al., 2023). The results suggest that our attentional processes and other salience networks are also tied into the pain neuromatrix. Emotions too will affect pain experiences (Caston et al., 2023). Seeing someone else react negatively to one’s injury will increase one’s own pain response, as will seeing someone else in pain (Wiech and Tracey, 2009; Budell et al., 2010; Bayet et al., 2014; Jauniaux et al., 2019). These effects appear throughout the spine and seem to reflect a separate modulatory system that evaluates environmental threats, which then facilitates or primes pain responses (Khatibi et al., 2023). To make matters even more complicated, vicarious pain and fear modulate self-pain responses differentially (*ibid.*), perhaps reflecting yet more different but overlapping networks connected to pain responses. As Caston et al. (2023) note, “brain dynamics can shift by changing just one aspect of the stimulus-perception-behavior relationship” (p. 14). These results hint at pain responses being dynamically adaptive.

Is pain processing highly multiplex? We are starting to find clues that it is. We know that pain processing is widely distributed across the brain and it interacts with and is impacted by other processing networks. As a result, pain experiences are context-dependent and highly individualized (Kucyi and Davis, 2014). The neuromatrix hypothesis implies that pain experiences are tied to synchronized activity across multiple distinct brain regions. However, it could also be the case that pain-sensitive neurons are locally intermingled with neurons that are less sensitive to pain-related information within areas. Given this, is the neural encoding of pain information carried in the brain at a coarse-grained regional level or at a fine-grained local level?

Put another way, we already know that virtually no brain areas are exclusively devoted to processing pain and nothing else (Mouraux et al., 2011; Liberati et al., 2016; Salomons et al., 2016; Su et al., 2019). Multivariate pattern analysis of fMRI data demonstrates that activation patterns in these areas differ between painful and non-painful stimuli, even when stimuli intensity is held constant (Liang et al., 2016).^4^ The question is surrounding the relative level of the pattern. Because fMRI data have limited spatial resolution, is it not clear whether these activity patterns are regionally based or locally based. Are there only pain-specific patterns across areas, or are there pain-specific neuronal responses within areas?

Recent studies suggest that the answer is both. Comparing global and regional multivariate pattern analyses of fMRI data for intensity-matched touch vs. pain stimuli, along with functional connectivity analyses between spatial scales, revealed pain-specific patterns at every level of analysis. Furthermore, the spatial distribution of pain-related processing was unique to individuals, which would explain individual variations in pain perception, pain vigilance, and pain expression (Wang S. et al., 2023). These data, of course, do not tell us that individual neurons are sensitive to pain as well as other stimuli. However, they do show that pain processing is strongly intermingled with other sorts of processing and that this intermingling occurs in all levels of organization thus far examined. Pain neurons might be multiplex, or the analyzed voxels could be multiplex, or both. The point is that the processing that underlies pain is not easily localized, nor does it appear to be devoted exclusively to pain and nothing else.

## 6 Conclusion: Everything, Everywhere, All at Once

If this way of understanding brain function is correct, then the concerns of Corns, Coninx, and others fall away, for their views on what theories of brain function might look like are mistaken. I agree with Corns that pain processes are not “mechanistic,” but I agree not because there is something different or special about pain but because no complex cognitive/emotional brain processes are mechanistic. Therefore, instead of concluding that differences in pain responses across individuals or over time belie scientific explanations of pain, we can see that such dynamic, heterogeneous, and multiplex responses likely represent normal brain functioning. With a different perspective on understanding brain functioning, it is no longer surprising that different neural structures in different locations across the brain can all somehow be involved in pain processing, but, at the same time, be individually neither necessary nor sufficient for the experience of pain. Eliminativism is not the only path forward.

It is also not scientifically damning that, as Coninx points out, multivariate pattern analyses of neuroimaging data for pain experiences are unique to individual subjects, pain type, and the larger psychosocial and emotional context. Understanding the brain in terms of dynamic, context-dependent, spatiotemporal trajectories would lead directly to this conclusion. At the same time, we perhaps need not abstract away individual differences among pain cases because we have (and are developing more) neuroscientific tools that allow for complex analyses of multiple variables interacting along multiple dimensions. Patience with scientific advancement might be a better strategy than using family resemblances to support only gross generalizations about pain experiences.

Finally, just as navigational challenges for animals are embedded in complex physical and sociocultural environments, so too is pain processing. Both require individualized brain responses. And just as animal navigation is fundamentally understood in terms of complex biopsychosocial trajectories of brain activity through a theoretical multidimensional space, so too are pain states. If a pain experience is the way that the brain dynamically responds to a particular combination of multidimensional biopsychosocial factors, as Oliver and others intimate, we could still have a very robust neuroscience of pain. This sort of complexity does not prevent a neural theory of pain. Affordance-based approaches could be encompassed in these new approaches.

The approach described herein would not reduce pain experiences, or even pain responses, in the way philosophers have traditionally assumed, but it would reflect the most theoretically grounded and analytically advanced perspectives of how brains work. In summary, pain is more than an unpleasant emotional and sensory experience, despite the IASP assertion to the contrary. While it may only be loosely associated with noxious stimuli, it is still a brain-based response to an animal’s internal or external environment. As such, it is something that neuroscientists can study in humans and in animal models. Furthermore, as our experimental and analytic techniques improve and grow ever more sophisticated, so too will our theories of pain processing. What comprises pain experiences is much more complicated and subtle than what philosophers have at least previously assumed. It is far, far too early to begin to throw in the towel and proclaim that a detailed understanding of pain as a brain function is beyond the pale. Our work here is only barely beginning.

The approach adumbrated herein is important not only conceptually but also practically, for it will shape how we treat and care for pain patients. Pain being more than just a sensory response, and bodily injury no longer being required for pain, opens the possibility of greater acceptance and more avenues of treatment for patients with historically dubious sorts of chronic pain, like fibromyalgia and chronic fatigue syndrome, as well as for things like menstrual pain, cancer pain, and other pains whose etiology we do not understand. We should also be able to better understand what nociplastic pain is and, therefore, how to treat it. Pain being essentially a whole-brain response that is integrated with other incoming and self-generated signals allows for nuance and differences across individual pains. The hope, my hope, is that this perspective will ultimately present ways to re-conceptualize the treatment of pain at a fundamental level.

## Author contributions

The author confirms being the sole contributor of this work and has approved it for publication.

## Footnotes

^1^Borg et al. (2021) and Coninx et al. (2023a,b) provide good arguments (and some empirical data) for why this position is fundamentally incoherent. I shall not dwell on this possibility here.

^2^See also Klein (2015) for another example of this approach to pain or Hall (2008) for an example of this approach to itch.

^3^Alpha-band event-related desynchronizations have been associated with cognitive and sensorimotor activity in cortex since at least the 1950s (e.g., Gastaut, 1952).

^4^See Iannetti et al. (2013) for a description of these techniques.
